# Association of cardiovascular health and epigenetic age acceleration

**DOI:** 10.1186/s13148-021-01028-2

**Published:** 2021-02-25

**Authors:** Tess D. Pottinger, Sadiya S. Khan, Yinan Zheng, Wei Zhang, Hilary A. Tindle, Matthew Allison, Gretchen Wells, Aladdin H. Shadyab, Rami Nassir, Lisa Warsinger Martin, JoAnn E. Manson, Donald M. Lloyd-Jones, Philip Greenland, Andrea A. Baccarelli, Eric A. Whitsel, Lifang Hou

**Affiliations:** 1 Center for Genetic Medicine, Northwestern University Feinberg School of Medicine, Chicago, IL USA; 2 Department of Preventive Medicine, Northwestern University Feinberg School of Medicine, Chicago, IL USA; 3 Division of Cardiology, Department of Medicine, Northwestern University Feinberg School of Medicine, Chicago, IL USA; 4 Division of General Internal Medicine and Public Health, Vanderbilt University Medical Center, Nashville, TN USA; 5 Geriatric Research Education and Clinical Centers (GRECC), Veterans Affairs Tennessee Valley Healthcare System, Nashville, TN USA; 6 Department of Family Medicine and Public Health, San Diego School of Medicine, University of California, La Jolla, CA USA; 7 University of Kentucky, Lexington, KY USA; 8 University of California Davis, Davis, CA USA; 9 George Washington University, Washington, DC USA; 10 Brigham and Women’s Hospital, Harvard Medical School, Boston, MA USA; 11 Columbia University, New York, NY USA; 12 Department of Epidemiology, Gillings School of Global Public Health, University of North Carolina, Chapel Hill, NC USA; 13 Department of Medicine School of Medicine, University of North Carolina, Chapel Hill, NC USA; 14 Institute for Genomic Medicine, Columbia University, 701 West 168th Street, New York, NY 10032 USA

**Keywords:** Cardiovascular health (CVH), Epigenetic age acceleration, Women’s health initiative (WHI), DNA methylation, Simple seven

## Abstract

**Background:**

Cardiovascular health (CVH) has been defined by the American Heart Association (AHA) as the presence of the “Life’s Simple 7” ideal lifestyle and clinical factors. CVH is known to predict longevity and freedom from cardiovascular disease, the leading cause of death for women in the United States. DNA methylation markers of aging have been aggregated into a composite epigenetic age score, which is associated with cardiovascular morbidity and mortality. However, it is unknown whether poor CVH is associated with acceleration of aging as measured by DNA methylation markers in epigenetic age.

**Methods and results:**

We performed a cross-sectional analysis of racially/ethnically diverse post-menopausal women enrolled in the Women’s Health Initiative cohort recruited between 1993 and 1998. Epigenetic age acceleration (EAA) was calculated using DNA methylation data on a subset of participants and the published Horvath and Hannum methods for intrinsic and extrinsic EAA. CVH was calculated using the AHA measures of CVH contributing to a 7-point score. We examined the association between CVH score and EAA using linear regression modeling adjusting for self-reported race/ethnicity and education. Among the 2,170 participants analyzed, 50% were white and mean age was 64 (7 SD) years. Higher or more favorable CVH scores were associated with lower extrinsic EAA (~ 6 months younger age per 1 point higher CVH score, *p* < 0.0001), and lower intrinsic EAA (3 months younger age per 1 point higher CVH score, *p* < 0.028).

**Conclusions:**

These cross-sectional observations suggest a possible mechanism by which ideal CVH is associated with greater longevity.

## Introduction

In 2010, the American Heart Association defined the construct of ideal cardiovascular health (CVH) as the simultaneous presence of 7 ideal health factors: healthy diet, absence of smoking, healthy body mass index (BMI), and optimal levels of physical activity, blood pressure, fasting glucose, and total cholesterol [1]. Higher levels of CVH have been prospectively associated with greater longevity and healthy longevity, as well as markedly lower incidence of chronic diseases related to aging [1, 2]. Higher CVH is also associated with lower incidence of cardiovascular disease [3], the leading cause of death among women in the United States [4]. Therefore, it is important to understand the biological mechanisms that may link CVH with favorable outcomes, which could assist novel biomarker discovery.

Epigenetic changes in patterns of DNA methylation are suggested as a promising mechanism, and epigenetic aging as an important surrogate biomarker, in the research of aging, mortality, and chronic disease risk [5]. Epigenetic clocks are methods that have been used to evaluate epigenetic changes that lead to deviations from chronological age, a concept known as epigenetic age acceleration. Previous studies have shown that accelerated epigenetic aging is predictive of poor health outcomes [6, 7]. Although epigenetic age acceleration has been associated with individual components of CVH [8], its relationship with CVH as a whole has not been established. In this study, we investigated the relationship between CVH and epigenetic age acceleration by leveraging an epidemiological cohort with objective assessment of CVH and blood DNA methylation data.

## Results

**Fig. 1:**
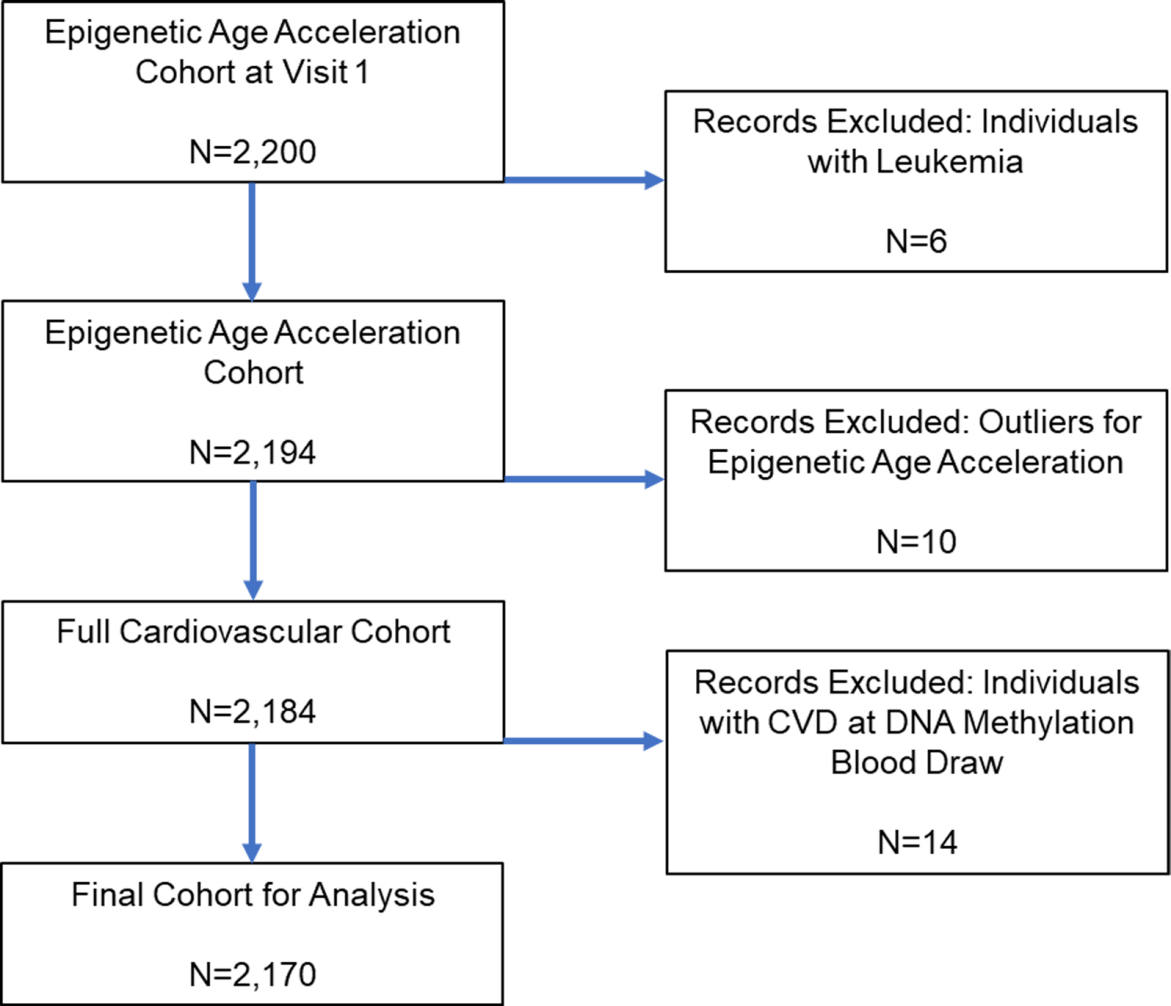
Flow diagram of individuals included for the analysis of cardiovascular health and epigenetic age acceleration

There were 2170 individuals who were included in these analyses after the exclusion of individuals due to leukemia diagnosis (*n* = 6), epigenetic age acceleration extremes (*n* = 10), and cardiovascular disease diagnosis (*n* = 14) (Fig. 1). Among the 2,170 WHI participants included in this study (Table 1), approximately 50% were non-Hispanic white, and most were non-smokers or quit smoking at least three-years prior to the blood draw. Proportionally, there were fewer non-Hispanic Black participants in the highest tertile of EEAA (faster aging) than lowest tertile, while there were more Hispanic participants in the highest tertiles of EEAA than the lowest tertile (*p* < 0.001, Table 1). In the highest tertile of IEAA, there were fewer participants with an ideal BMI than the lowest tertile (Additional file 1: Table 1). This difference was, however, not statistically significant.

**Table 1:** Extrinsic epigenetic age acceleration cohort characteristics

	Overall	EEAA T1 (< − 2.24 years.)^Ref^	EEAA T2 (− 2.24–2.33 years.)	EEAA T3 (> 2.33 years.)
N	2170	724	727	719
*Race/ethnicity (%)*				
Non-Hispanic Black or African-American	554 (25.53)	252 (34.81)	178 (24.48)***	124 (17.25)***
Hispanic/Latino	315 (14.52)	62 (8.56)	117 (16.09)***	136 (18.92)***
Non-hispanic white	1083 (49.91)	356 (49.17)	352 (48.42)	375 (52.16)
Other	218 (10.05)	54 (7.46)	80 (11)*	84 (11.68)**
Chronological age years (sd.)	64.19 (7.06)	64.35 (7.11)	64.05 (7.09)	64.19 (7)
*Education (%)*				
Less than high school	198 (9.2)	55 (7.66)	61 (8.46)	82 (11.5)
High school diploma or GED	395 (18.36)	108 (15.04)	141 (19.56)*	146 (20.48)**
Vocational, some college, associates	848 (39.41)	283 (39.42)	295 (40.92)	270 (37.87)
College degree or greater	711 (33.04)	272 (37.88)	224 (31.07)**	215 (30.15)**
*Components of CVH score (%)*				
Non-smoking	1,880 (87.81)	634 (88.55)	626 (87.31)	620 (87.57)
Ideal BMI	521 (24.13)	180 (25.07)	188 (25.9)	153 (21.4)
Ideal physical activity	428 (21.59)	150 (22.9)	153 (22.9)	125 (18.97)
Ideal cholesterol levels	1,536 (79.22)	505 (79.03)	514 (78.47)	517 (80.16)
Ideal glucose levels	1,928 (88.85)	657 (90.75)	640 (88.03)	631 (87.76)
Ideal blood pressure	414 (19.08)	147 (20.3)	133 (18.29)	134 (18.64)
Ideal diet	606 (27.93)	205 (28.31)	201 (27.65)	200 (27.82)
*CVH score (%)*				
0–1	41 (2.16)	13 (2.07)	16 (2.48)	12 (1.91)
2	213 (11.2)	59 (9.39)	89 (13.82)**	65 (10.33)
3	636 (33.46)	213 (33.92)	188 (29.19)	235 (37.36)
4	574 (30.19)	186 (29.62)	207 (32.14)	181 (28.78)
5	294 (15.47)	104 (16.56)	94 (14.6)	96 (15.26)
6–7	143 (7.52)	53 (8.44)	50 (7.76)	40 (6.26)

Ref: reference group (T2/3 compared to T1); *p value < 0.05; ** p value < 0.01; *** p value < 0.001

BMI: body mass index; GED: general education diploma; CVH: cardiovascular health

Non-smoking: non-smokers or individuals who quit smoking three year prior to blood draw

Ideal cholesterol levels: cholesterol below 200 without medication

Ideal glucose levels: glucose below 100 without medication

Ideal BMI: BMI below 25

Ideal physical activity: greater than 150 min of moderate to strenuous activity or 75 min of strenuous activity

Ideal blood pressure: systolic blood pressure equal to or below 120, diastolic blood pressure below 80 without hypertension medication

Ideal diet: assessed based on calcium, potassium, fiber, and saturated fat intake

Participants with higher (more favorable) CVH score had lower EEAA (~ 6-month lower age per 1 point higher CVH score; *p* < 0.0001, Table 2). This association was consistent when analyzing IEAA (~ 3-month lower age per 1 point higher CVH score, *p* < 0.028, Table 2). For EEAA, a similar direction of association was seen when using the 3-level and 14-point definitions of CVH, with the 14-point definition showing a 2-month lower age per 1 point higher CVH (*p* < 0.0035, Additional file 1: Table 2). The 3-level definition of CVH, however, was not significantly associated with EEAA in this analysis (*p* > 0.05, Additional file 1: Table 2). Additionally, for IEAA, the alternative definitions were not significant (both *p* > 0.05, Additional file 1: Table 2).

**Table 2:** Results of regression analysis of extrinsic and intrinsic epigenetic age acceleration on ideal health score among participants free of cardiovascular disease at DNA methylation blood draw

	β	Standard error	*p* value
*EEAA*			
Cardiovascular Health Score (7-point)	− 0.462 (years)	0.118	< 0.0001
*IEAA*			
Cardiovascular Health Score (7-point)	− 0.209 (years)	0.0956	0.0283

EEAA: extrinsic epigenetic age acceleration; IEAA: intrinsic epigenetic age acceleration

Results are adjusted for self-reported race/ethnicity and education

## Discussion

In this study, we estimated the association between CVH at middle and older age and contemporaneous indicators of epigenetic age acceleration, leveraging data from 2170 post-menopausal women selected from the Women’s Health Initiative. We found that higher or more favorable CVH scores were associated with a lower extrinsic and intrinsic age acceleration among individuals without CVD. When multiple approaches to assess and quantify CVH were evaluated, the association between CVH and EEAA was consistent and similar, however, the association between CVH and IEAA was no longer significant. The loss of a statistical association between CVH and IEAA for 3-level and 14-point scale model could be due to the change of the granularity of the data points that lead to a change in the linearity of the relationship. These data suggest that poor CVH could be acting through DNA methylation mechanisms to accelerate aging.

Previous studies have shown that factors similar to those included in the definition of CVH as well as the covariates used in these analyses, such as diet, physical activity, education and metabolic syndrome, are associated with epigenetic changes [8, 9]. The magnitude of association is also comparable to previous studies [9]. While a healthy diet that includes regular consumption of fish, fruits, and vegetables showed a relatively weak correlation with lower epigenetic aging, high education and high physical activity did have an association with lower extrinsic epigenetic age acceleration [8]. In addition, high body mass index, high triglycerides, and high blood pressure are associated with higher extrinsic and intrinsic epigenetic age acceleration [8, 10]. Since these outcomes are associated with cardiometabolic changes, these data suggest that changes epigenetic age acceleration may be associated with cardiovascular outcomes [11]. However, similar to our study, these prior studies were cross-sectional, and the temporality of association is unclear.

High CVH is known to be associated with lower mortality for many outcomes including cardiovascular disease [3]. Epigenetic age acceleration has been shown to be associated with an increase in cardiovascular disease mortality and all-cause mortality [6]. Conceptually, intrinsic epigenetic age is thought to be stable and not changed by external changes in the environment such as behavior and lifestyle changes and is often more related to chronological aging. Conversely, extrinsic epigenetic age is thought to be more influenced by environmental and lifestyle factors [12]. The strong association of CVH with extrinsic epigenetic age acceleration that we observe in these analyses could be affected by the lifestyle factors that are used to calculate the CVH scores. However, precise quantification of diet and physical activity is difficult. Therefore, extrinsic epigenetic age acceleration could be a more sensitive biomarker for CVH than intrinsic epigenetic age acceleration.

The strength of this study includes the availability of DNA methylation data on a moderately to large-sized sample of women. Additionally, we analyzed CVH utilizing the AHA’s “Life’s Simple 7” ideal lifestyle and clinical factors as a single metric rather than using the individual components as many of these factors are correlated. Limitations include lack of generalizability of the results to men or younger women. We were able to analyze measures that were temporally proximate to those of DNA methylation age acceleration. However, as this is a cross-sectional design, causality could not be established. Future studies could consider the amount of time that would be adequate to observe epigenetic changes after positive lifestyle changes. In addition, DNA methylation clocks present challenges for integrating values for clinical utility which could be improved by increasing the population size of the training data used to make inferences as well as increase the ancestral diversity of these data [13, 14]. Furthermore, it is difficult to assess causality between DNA methylation age acceleration and outcome. As such, additional methods to establish causal relationships have been utilized to explore the causality [15, 16].

## Conclusions

This research shows an association between CVH and epigenetic age acceleration. This work provides initial evidence for epigenetic age acceleration to be considered as a potential early detection biomarker for CVH. Future studies are needed to evaluate if measures to promote optimal CVH through lifestyle and behavioral interventions could substantially alter an individual’s epigenetic signature and the clinical utility of such a signature. In future, epigenetic data have the possibility to provide novel information that could be used in addition to genetic information, such as polygenic risk scores, to refine prediction for an individual’s risk for cardiovascular disease.

## Methods

### Study population

The Women’s Health Initiative (WHI) is a cohort of post-menopausal women who were enrolled from 1993 to 1998 and were initially between the ages of 50 and 79 years. Inclusion and exclusion criteria for participation in the WHI study have been described [17]. From the overall WHI cohort, a stratified, racially/ethnically diverse sample of 2200 WHI clinical trial participants with available stored serum were selected for DNA methylation analysis at either the screening visit or annual visits [15]. A description of the clinical trials has been published [17]. Briefly, data were collected on participant demographics, and health outcomes through questionnaire and clinical examination on initial and follow-up visits. Baseline blood samples were collected from participants in EDTA tubes after an overnight fast. These specimens were stored at – 70 °C and processed by the WHI core laboratories [8]. Data used for this analysis were from the closest visit date preceding or on the date of blood draw for DNA methylation analysis (64% of which were on the same day, 30% within 2–5 years, and 6% within 5–7 years earlier).

### Measurements

#### Outcome

*DNA Methylation Data.* DNA methylation assays were performed using the Illumina Infinium Human-Methylation450 Bead Chip [18] and was conducted at the HudsonAlpha Institute of Biotechnology. Genome-wide DNA methylation data was used to estimate epigenetic age (DNAm) using the proportion of modified signal at each CpG site. Horvath’s [19] and Hannum methods [20] were used to calculate intrinsic and extrinsic epigenetic age acceleration, respectively. Intrinsic epigenetic age acceleration (IEAA) is a residual value calculated from regressing DNAm age using 353 CpGs on blood cell counts of naïve and exhausted CD8 + T cells, CD4 + T cells, plasma B cells, natural killer cells, monocytes, and granulocytes [21]. Extrinsic epigenetic age acceleration (EEAA) was calculated using DNAm age using 71 CpGs, and cell counts of naïve and exhausted cytotoxic T cells, and plasma B cells- cell types that are known to be associated with age. A linear regression model that regressed on the weighted estimated age was used to generate residual values of acceleration [8]. Methylation data from an individual with a leukemia diagnosis would introduce bias in the methylation analyses. Therefore, individuals with a leukemia diagnosis were removed from these analyses.

#### Exposure: cardiovascular health score

The CVH score was calculated based on the American Heart Association “Life’s Simple Seven” measures for CVH [1]. These seven factors include diet, physical activity, smoking status, BMI, blood pressure, fasting glucose, and total cholesterol. The clinical and behavioral methods that contribute to CVH were defined, scored, and summed to create an ideal cardiovascular health score as in Foraker, et al. [2]. An individual was assigned a score of 1 for each ideal value for the category described generating a minimum score of 0 and a maximum score of 7. For example, if an individual had an ideal glucose level, a fasting glucose of less than 100 without medication, they were assigned a score of 1 for ideal glucose. An individual with a fasting glucose level greater than 100, or an individual who received treatment for diabetes, was assigned a score of 0. Ideal values for each category can be found in the footnote of Table 1, Additional file 1: Fig. 1, and Table 1. Individuals with a score of 0 or 1 were combined into a single stratum, as were individuals with a score of 6 or 7. Individuals with a higher score had better or more favorable cardiovascular health. Sensitivity analyses were performed to include other definitions of CVH including a 14-point definition in which 0 to 2 points were assigned to each of the “Life’s Simple 7” metrics (0 for poor, 1 for intermediate, 2 for ideal) and treated as continuous, and a 3-level definition which is a collapsed version of the 14-point score (low = 1–7; intermediate = 8–11; high = 12–14).

### Statistical analysis

To describe baseline characteristics and CVH score, the cohort was divided into tertiles of EEAA/IEAA. The CVH 7-point score metric was used as the exposure variable to test for relationships with intrinsic and extrinsic age acceleration in a multivariable linear regression model, adjusting for self-reported race/ethnicity and education. Values for covariates were calculated before or at the time of blood draw. Sensitivity analyses were conducted using the 3-level definition of CVH and the 14-point definition of CVH in a multivariable linear regression model, adjusting for self-reported race/ethnicity and education. Individuals with values of epigenetic age acceleration greater than 25 or less than – 25 were excluded in these analyses to reduce the statistical deviations. The software package SAS 9.4 was used for this analysis.

## Supplementary Information

**Additional file 1:** Supplemental Figures and Tables.

## Data Availability

The WHI data are available through dbGAP: http://www.ncbi.nlm.nih.gov/projects/gap/cgi-bin/study.cgi?study_id=phs000200.v10.p3.
