# Supplementary material for: Association of cardiovascular health and epigenetic age acceleration
Source: Clin Epigenetics. 2021 Feb 25;13:42. doi: 10.1186/s13148-021-01028-2 (PMC7905851; doi:10.1186/s13148-021-01028-2)
Supplement: Supplementary file 1 — Additional file 1: Supplemental Figures and Tables. [file 13148_2021_1028_MOESM1_ESM.docx]

**SUPPLEMENTAL FIGURES**


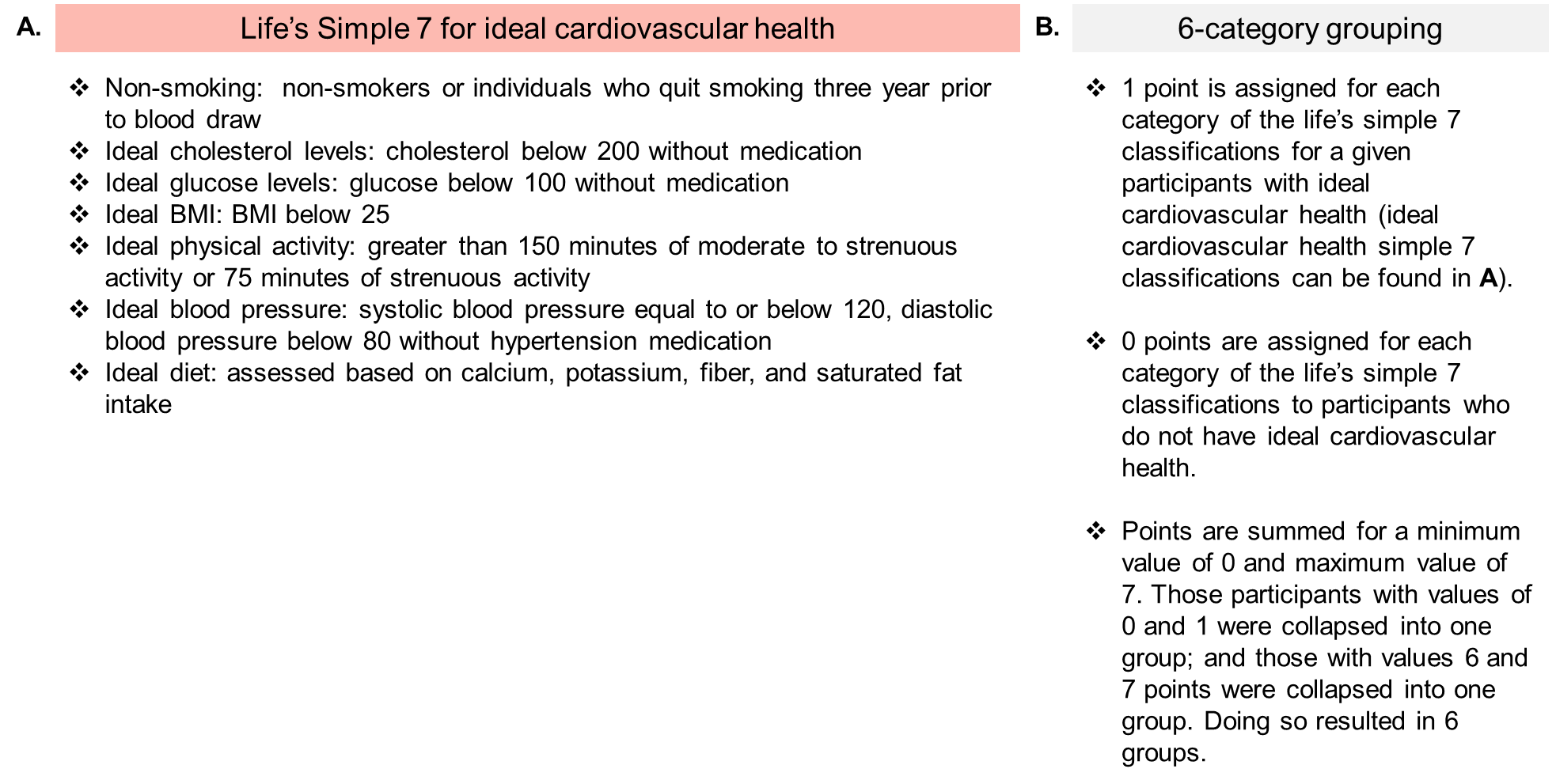


**Supplemental Figure 1. Methods used for categorizing participants**. **A**. Utilizing the American Heart Association’s Life’s Simple 7 classifications, thresholds were used to categorize participants into ideal cardiovascular health groupings. **B**. Participant were assigned points according to ideal cardiovascular health status as determined by the American Heart Association’s Life’s Simple 7 in **A**.

**SUPPLEMENTAL TABLES**

**Supplemental Table 1.** Intrinsic epigenetic age acceleration cohort characteristics

|  | **Overall** | **IEAA T1**  **(< -1.96 yrs.)^Ref^** | **IEAA T2**  **(-1.96 – 1.83 yrs.)** | **IEAA T3**  **(> 1.83 yrs.)** |
| --- | --- | --- | --- | --- |
| **N** | 2,170 | 725 | 726 | 719 |
| **Race/Ethnicity (%)** |  |  |  |  |
| Non-Hispanic Black or African-American | 554 (25.56) | 212 (29.24) | 160 (22.04)** | 182 (25.31) |
| Hispanic/Latino | 315 (14.51) | 117 (16.14) | 130 (17.91) | 68 (9.46)*** |
| Non-Hispanic White | 1,083 (49.88) | 328 (45.24) | 362 (49.86) | 393 (54.66)*** |
| Other | 218 (10.04) | 68 (9.38) | 74 (10.19) | 76 (10.57) |
| **Chronological Age Yrs (sd.)** | 64.19 (7.06) | 64.37 (7.16) | 64.12 (7.11) | 64.09 (6.91) |
| **Education (%)** |  |  |  |  |
| Less than high school | 198 (9.2) | 69 (9.62) | 73 (10.14) | 56 (7.83) |
| High school diploma or GED | 395 (18.35) | 122 (17.02) | 139 (19.31) | 134 (18.74) |
| Vocational, some college, associates | 849 (39.43) | 268 (37.38) | 293 (40.69) | 287 (40.14) |
| College degree or greater | 711 (33.02) | 258 (35.98) | 215 (29.86)* | 238 (33.29) |
| **Components of CVH Score (%)** |  |  |  |  |
| Non-smoking | 1,881 (87.82) | 622 (87.24) | 632 (87.9) | 626 (88.29) |
| Ideal BMI | 521 (24.12) | 188 (26) | 172 (23.86) | 161 (22.52) |
| Ideal Physical Activity | 428 (21.58) | 143 (21.5) | 145 (21.9) | 140 (21.37) |
| Ideal Cholesterol Levels | 1,537 (79.23) | 527 (81.83) | 497 (76.23) | 512 (79.63) |
| Ideal Glucose Levels | 1,929 (88.85) | 645 (88.97) | 639 (88.02) | 645 (89.57) |
| Ideal Blood Pressure | 414 (19.07) | 142 (19.59) | 138 (19.01) | 134 (18.64) |
| Ideal Diet | 606 (27.91) | 209 (28.83) | 212 (29.2) | 185 (25.73) |
|  |  |  |  |  |
| **CVH Score (%)** |  |  |  |  |
| **0-1** | 41 (2.16) | 12 (1.9) | 17 (2.66) | 12 (1.91) |
| **2** | 213 (11.2) | 62 (9.81) | 78 (12.19) | 73 (11.61) |
| **3** | 637 (33.49) | 211 (33.39) | 202 (31.56) | 223 (35.45) |
| **4** | 574 (30.18) | 197 (31.17) | 189 (29.53) | 188 (29.89) |
| **5** | 294 (15.46) | 97 (15.35) | 101 (15.78) | 96 (15.26) |
| **6-7** | 143 (7.52) | 53 (8.39) | 53 (8.28) | 37 (5.88) |

Ref: reference group (T2/3 compared to T1); * p-value <0.05; ** p-value <0.01; *** p-value <0.001

BMI: body mass index; GED: general education diploma; CVH: cardiovascular health

Non-smoking: non-smokers or individuals who quit smoking three year prior to blood draw

Ideal cholesterol levels: cholesterol below 200 without medication

Ideal glucose levels: glucose below 100 without medication

Ideal BMI: BMI below 25

Ideal physical activity: greater than 150 minutes of moderate to strenuous activity or 75 minutes of strenuous activity

Ideal blood pressure: systolic blood pressure equal to or below 120, diastolic blood pressure below 80 without hypertension medication

Ideal diet: assessed based on calcium, potassium, fiber, and saturated fat intake

**Supplemental Table 2.** Results of regression analysis on ideal health score for extrinsic and intrinsic epigenetic age acceleration (3-strata and 14-point approaches) among participants free of cardiovascular disease at DNA methylation blood draw

|  | **β** | **Standard Error** | **P-value** |
| --- | --- | --- | --- |
|  |  |  |  |
| **EEAA** |  |  |  |
| Ideal Health Score (14-point) | -0.174 (yrs.) | 0.0595 | 0.0035 |
| Ideal Health Score (3-strata) | -0.0274 (yrs.) | 0.0143 | 0.055 |
|  |  |  |  |
| **IEAA** |  |  |  |
| Ideal Health Score (14-point) | -0.0484 (yrs.) | 0.04817 | 0.31 |
| Ideal Health Score (3-strata) | -0.0008 (yrs.) | 0.0167 | 0.96 |
|  |  |  |  |

EEAA: extrinsic epigenetic age acceleration; IEAA: intrinsic epigenetic age acceleration

Results are adjusted for self-reported race/ethnicity and education
